# Complementary medicine use by the Australian population: a critical mixed studies systematic review of utilisation, perceptions and factors associated with use

**DOI:** 10.1186/s12906-016-1143-8

**Published:** 2016-06-11

**Authors:** Rebecca Reid, Amie Steel, Jon Wardle, Andrea Trubody, Jon Adams

**Affiliations:** Australian Research Centre in Complementary and Integrative Medicine, University of Technology, Sydney, Ultimo, NSW 2007 Australia; Endeavour College of Natural Health, 269 Wickham St, Fortitude Valley, QLD 4006 Australia

**Keywords:** Complementary medicine, Health services use, Chronic disease, Sociodemographics, Sociological factors

## Abstract

**Background:**

There is increasing evidence that complementary medicine (CM) services are being used by a substantial proportion of the Australian population and this topic has attracted keen interest from primary health care providers and policy makers. This article outlines the first summative critical review of the predictors of CM use in Australia as well as the characteristics and perceptions of Australian CM users over the last 14 years.

**Methods:**

A literature search was conducted to ascertain original research from 2000 to 2014 in the AMED; CINAHL; and PubMed databases. Selected articles were subject to a critical appraisal analysis to identify the quality of the article. The search was confined to peer reviewed original articles published in English which identified the nature of CM services use in Australia.

**Results:**

The findings indicate a correlation between CM users and gender, with reports of a higher rate of use from females compared to males. Female CM users are more likely to be middle-aged with a higher education and higher annual income in comparison to female non-CM users. An association between resident location and use of CM disciplines was also identified with reports of rural residents utilising manual therapies more frequently compared to urban residents. CM users are more likely to seek CM services for a range of chronic conditions including diseases identified as National Health Priority Areas by the Australian Government.

**Conclusions:**

This article provides the first comprehensive review examining the nature of CM use in Australia. The review findings offer important insights into the characteristics and features of CM use in Australia and provide insights for national and regional primary health care initiatives and of interest to medical doctors, allied health professionals, CM practitioners, researchers and policy makers.

## Background

Complementary medicine (CM) refers to a diverse collection of clinical practices (such as acupuncture, massage therapy and naturopathy) and treatments (such as herbal medicine and homeopathy) not traditionally associated with the conventional medical curriculum [1]. Australia is one country in which CM use is particularly significant with some of the highest CM utilisation in the developed world [2]. Coupled with high utilisation is a high CM practitioner population which outnumbers conventional medical providers in some areas [3].

The increasing use of CM services by the general population has gradually resulted in CM becoming an important subject amongst Australian primary health care professionals and policy makers. Most recently, the Federal Department of Health and Aging (DOHA) commissioned a review on the Australian Government Rebate on Private Health Insurance for Natural Therapies [4]. In addition, Australia’s National Health and Medical Research Council (NHMRC) have published a statement to assist health consumers in Australia in making informed decisions regarding their health care including a close scrutiny of the evidence associated with CM [5]. Whilst some CM professions and products are regulated by governing bodies in Australia, often at levels beyond that observed in other countries, most CM provision remains informal or unregulated, and is generally not integrated into conventional health care frameworks [6].

Alongside the attention given to CM by Australian policy makers, a number of other issues have been identified as significant concerns within the Australian health system. One such issue is the growing prevalence of chronic disease and the associated pressure this places on health spending in Australia [7], as highlighted in the National Health Priority Areas (NHPA) [8]. Another issue is the need to strengthen primary health care services due to a number of concerns related to accessibility amongst high risk populations including individuals with chronic disease [9]. Rurality is also a factor which attracts ongoing attention within Australian health policy due to the large rural and remote areas in Australia and the impact this has on the ability to provide timely and quality health care appropriate to the needs of the population [10].

International data from the USA, UK, Norway and Canada identifies key demographic and health related factors which have been recognised as independent predictors for CM use in the general population. Compared to non-CM users, CM users are: more likely to be female and middle-aged; [11–13] have higher levels of income and education; [12–14] have multiple health concerns or diseases [12]; and reside in a non-urban area [15]. However the characteristics of CM users are not always consistent in all countries possibly due to local or regional political, social and economic factors [2]. Various ‘push’ and ‘pull’ factors, defined as the positive or negative motivations regarding CM use respectively, have also been identified as influential in driving CM use in some populations. Examples of ‘pull factors’ include dissatisfaction with conventional care and concerns about the safety of pharmaceutical medication whilst ‘push factors’ include alignment with personal beliefs, attraction of the holistic principles of CM or desire for greater personal control of their wellbeing [16].

Given the growing prevalence of CM use in Australia, there is a need for policy makers and practitioners to respond meaningfully to this component of the Australian healthcare system [17]. Developing a considered, co-ordinated response to CM use requires a clear understanding of the nature of CM use including the characteristics of users, the details of CM use and patients’ motivations for using CM. In response, this article reports findings from the first comprehensive and rigorous critical review of the current contemporary literature reporting original empirical research on the profile of Australian CM users, the CM services being used and the reasons for CM use amongst the Australian population.

## Methods

A database search was conducted to identify peer-reviewed original research published from January 1 2000 to December 31 2014 investigating the nature of CM use amongst the Australian population. The search included the following databases: AMED; CINAHL; and PubMed. The search terms employed were: *complementary medicine*; *alternative medicine; natural medicine; herbal medicine; complementary therapies; traditional medicine; holistic health; phytotherapy; naturopathy; supplements; acupuncture; massage; yoga; aromatherapy; homeopathy* and *Australia*. The following search strategy was used within all search fields in PubMed: (Australia) AND [(complementary medicine) OR alternative medicine) OR aromatherapy) OR natural medicine) OR yoga) OR herbal medicine) OR supplements) OR acupuncture) OR naturopathy) OR massage) OR complementary therapies) OR holistic health) OR homeopathy) OR traditional medicine) OR phytotherapy)]. Manual searching was also conducted to ensure known relevant articles were included in the review. All articles were imported into Mendeley, a bibliographic management software system and analysed based on title, abstract and full text. Articles were included if they reported peer-reviewed original research findings from new empirical data collection reporting on CM use in Australia, whilst articles were excluded if they were commentaries, editorials or literature reviews and were non-English. The database search was supplemented by an internet search using the same search terms as above, to identify any additional items, and bibliographic searching of included materials was also used to identify additional material. One author conducted the search and downloaded the results into Mendeley. Two authors independently examined the title and abstract of each result to identify relevant studies for inclusion. This review employed a mixed methods approach [18].

### Critical appraisal analysis

A critical appraisal quality research tool was used to examine the validity and worth of the selected articles. The critical appraisal tool was designed to compare and evaluate the studies based on a scoring system which identified three dimensions: the methodology, participant characteristics and definition of CM. The criteria listed is equally weighed with one point being allocated to each criteria. This appraisal score has been modified from previous mixed method reviews [19, 20] and has been designed to allow for different study designs to be compared equally based on their total score. This tool has been described elsewhere [19, 20]. Methodology assessment was based on representative sampling method, a sample size >500, response rate >75 % and a low recall bias. Critical evaluation of participant characteristics was appraised according to age, gender, residence location, socioeconomic status and health status (relating to CM use in chronic disease). Finally, studies were appraised for inclusion of the researcher’s definition of CM. Each aspect of the three dimensions were given 1 point if the paper identified the minimum requirement and a final score was tallied with a maximum potential score of 10.

Categorical grouping of the identified articles was also conducted. This process involved reading and re-reading the articles and extracting relevant data to categorise common themes identified in the literature. A common theme was defined by the authors as a topic or characteristic that appeared consistently across a number of the selected articles. Other critical integrative reviews identify and develop themes in the same manner, by assessing their findings and identifying consistency on a particular topic or characteristic that holds relevancy to the research topic [19, 21–25]. Once themes were identified, articles were allocated to appropriate categories with each article allocated to as many categories as was relevant. Categorical grouping of themes allowed for contrast and comparison of reported findings within the identified articles.

### Risk of bias assessment

All selected articles underwent a risk of bias assessment utilising an existing tool used to assess the risk of bias in prevalence studies. This tool identifies 4 main domains of bias including external validity, internal validity, measurement bias, and bias relating to analysis. The tool is comprised of 10 items and includes a summary assessment and is described elsewhere [26].

## Results

**Fig. 1:**
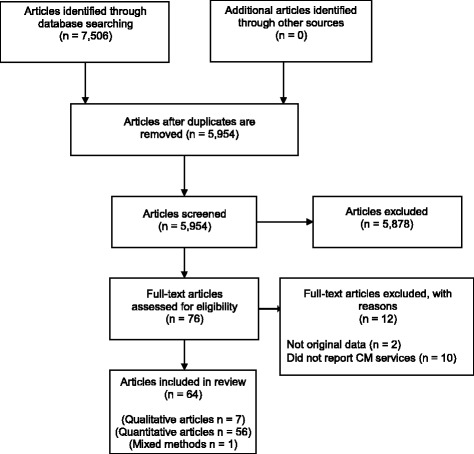
Flowchart outlines the methodological process of selection of articles included in the review

A total of 64 articles were selected for review between 2000 and 2014, with a majority published between 2007 and 2014. Of the selected articles, 56 employed quantitative which comprised of 17 longitudinal studies and 39 cross sectional surveys. Seven articles utilised qualitative research methods which included, two focus group studies, two structure interview studies, two semi-structured interview studies and one interview (design not-specified). Only one study utilised mixed methods. Figure 1 summarizes the7 literature search process.

Most studies were national (*n* = 23) in scope whilst others focused on specific geographic regions including South Australia (*n* = 8), Queensland (*n* = 4), Victoria (*n* = 14) and New South Wales (*n* = 15). The critical appraisal analysis recognised 9 articles with a score of 9. Significant gaps were identified in the selected articles with particular reference to the methodology of the studies, with approximately two thirds of the articles not reporting adequate representative sampling methods or a response rate of 75 % or above. A minority of articles provided a discrete definition of CM, which was defined as the definition of CM that was applied in their research. The minimum requirement to meet this criteria was that the researchers clearly stated what they meant by CM. In many cases such a definition was not provided. In particular, definitions where CM was only described as treatments not specifically recommended or prescribed by a doctor were considered to not to fulfil this criteria. An example of a CM definition used was: consultations with a CM practitioners [27–29]. However, the definition of CM was not consistent across articles or employed frequently enough to be of value. Results from the critical analysis tool is displayed in Table 1.

**Table 1:** Critical appraisal tool analysis results

Quality assessment	Methodology				Participant characteristics					CM definition	Score
	Representative sampling method	Sample size >500	Response rate >75 %	Low recall bias	Age	Gender	Residence location	Socioeconomic status	Health status		
Adams et al., 2003 [27]	X	X		X	X	X	X	X	X	X	9
Adams et al., 2005 [75]	X	X		X	X	X	X		X	X	8
Adams et al., 2007 [47]	X	X	X	X	X	X	X	X	X		9
Adams et al., 2011 [49]	X	X		X	X	X	X	X	X		8
Adams et al., 2012 [55]	X	X		X	X	X			X		6
Adams et al., 2013 [73]	X	X	X	X	X	X	X		X		8
Alderman & Kiepfer, 2003 [86]				X	X	X			X		4
Basedow et al., 2014 [46]	X		X	X	X	X		X	X		7
Braun & Cohen, 2011 [59]			X	X		X		X			4
Broom et al., 2012a [68]	X	X		X	X	X			X		6
Broom et al., 2012b [77]	X	X		X	X	X			X		6
Brownie, 2006 [71]	X	X	X	X	X	X		X	X		8
Buchbinder et al., 2002 [72]	X			X	X	X		X	X		6
Canaway & Manderson, 2013 [28]	X	X		X	X	X	X	X	X	X	9
Chatfield et al., 2009 [80]			X	X	X	X	X	X	X	X	8
Correa-Velez et al., 2003 [65]			X	X	X	X		X	X	X	7
Correa-Velez et al., 2005 [66]	X			X	X	X			X		5
D’Onise et al., 2013 [31]	X	X		X	X	X		X	X	X	8
Day, 2002 [82]			X	X	X				X		4
Day et al., 2004 [39]				X	X	X			X		4
De Visser et al., 2000 [56]		X			X	X		X	X		5
Dunning, 2003 [38]				X	X	X			X		4
Edwards et al., 2014 [35]		X		X	X	X			X		5
Feldman & Laura, 2004 [44]		X			X				X		3
Field et al., 2008 [57]		X	X		X	X	X	X	X		7
Fong & Fong, 2002 [43]					X	X			X		3
Forster et al., 2006 [70]		X	X	X	X	X	X	X	X		8
Frawley et al., 2013 [29]	X	X	X	X	X	X		X	X	X	9
George et al., 2004 [60]				X	X	X				X	4
Gollschewski et al., 2004 [87]	X	X		X	X	X	X	X	X		8
Heath et al., 2012 [69]				X	X	X			X		5
Hunter et al., 2014 [37]			X	X	X	X		X	X		6
Klafke et al., 2012 [36]			X	X	X	X		X	X	X	7
Kremser et al., 2008 [53]				X	X	X		X	X		5
Leong et al., 2009 [84]				X		X	X	X	X		5
Lim et al., 2005 [42]		X		X	X	X			X	X	6
MacLennan et al., 2006 [34]		X		X	X	X	X	X	X		7
Magin et al., 2006 [63]				X	X	X			X		4
Mak & Faux, 2010 [52]				X	X	X		X	X	X	6
Markovic et al., 2006 [51]					X	X			X	X	4
Murthy et al., 2014a [74]	X	X	X	X	X	X	X	X	X		9
Murthy et al., 2014b [95]	X	X	X	X	X	X	X	X	X		9
O’Callaghan& Jordan, 2003 [96]				X	X	X		X			4
Patching van der Sluijs, et al., 2007 [50]		X			X	X		X	X		5
Rayner et al., 2009 [54]					X	X			X		3
Sarris et al., 2010 [76]	X				X	X		X	X		5
Shenfield et al., 2002 [58]					X				X		2
Shorofi & Arbon, 2010 [45]				X	X	X	X	X			5
Sibbritt et al., 2006 [79]	X	X	X	X	X	X	X	X	X		9
Sibbritt et al., 2013 [62]	X	X		X	X	X	X	X	X		8
Sinha & Efron, 2005 [40]				X	X	X			X		4
Skouteris et al., 2008 [97]				X	X	X		X	X		5
Smith & Eckert, 2006 [67]	X	X		X	X	X		X	X		7
Smith et al., 2013 [85]			X	X	X	X		X	X		6
Spinks et al., 2014 [83]		X	X	X	X	X	X	X	X		8
Stankiewicz et al., 2007 [48]				X	X	X		X			4
Steel et al., 2012 [64]	X	X	X	X	X	X		X	X		8
Steel et al., 2014a [98]	X	X	X	X	X	X	X	X	X		9
Steel et al., 2014b [99]	X	X	X	X	X	X	X	X	X		9
Trutnovsky et al., 2001 [78]				X	X	X			X		4
Wadhera et al., 2011 [41]				X	X	X		X	X	X	6
Wilkinson & Simpson, 2001 [32]	X			X	X	X	X	X	X		7
Wilkinson & Jelinek, 2009 [33]				X	X	X	X	X	X		5
Xue et al., 2007 [30]	X	X		X	X	X	X	X	X		8

Articles reported four broad areas: the socio-demographic characteristics of CM users; health service utilisation of CM users; drivers of CM use and CM use in health subpopulations. Categorical grouping of the selected articles is presented in Table 2.

**Table 2:** Results and categorical grouping displaying the socio-demographic characteristics of CM users (1); Drivers of CM use (2); health service utilisation of CM users (3) and (4) CM use amongst health subpopulations

Author	Method	Target population	Sample (n)	Appraisal score	Results	Themes			
						1	2	3	4
Adams et al., 2003 [27]	Longitudinal study	Women	41,817	9	Higher CM use by non-urban women. CM use in older women used CM in conjunction with medication for chronic disease. >97 % consulted with a CM practitioner.	X			X
Adams et al., 2005 [75]	Longitudinal study	Middle age women	11,202	8	15.7 % cancer patients consulted with a naturopath/herbalist. CM users consulted with both CM & conventional practitioners. CM users were more likely rural residents & have school education only (49 %).	X		X	X
Adams et al., 2007 [47]	Longitudinal survey	Middle aged women	11,202	9	8.7 % women consulted with a naturopath, 1.4 % consulted with an herbalist. CM users more likely in non-urban areas (63 %) compared to 37 % in urban areas. Women who used naturopath also used conventional practitioners more frequently.	X		X	
Adams et al., 2011 [49]	Longitudinal study	Middle aged women	10,638	8	Women who consulted with a CM practitioner experienced more symptoms. Women with diploma or university education use CM more than non-CM users & more likely to reside in urban areas. No difference in consultation numbers between CM users & non-CM users for chiropractic, osteopathy, acupuncture & naturopathy.	X		X	
Adams et al., 2012 [55]	Longitudinal study	Self-reported depression	7,164	6	62 % of women used both conventional practitioners & CM (chiropractor 18 %, osteopathy 7 %, massage therapy 44 %, acupuncture 9 %, & naturopath 22 %).			X	X
Adams et al., 2013 [73]	Longitudinal study	Middle aged women	1,800	8	63.9 % consulted with a massage therapist, 43 % a chiropractor, & 22.9 % naturopath. Women in rural & outer regional areas used chiropractors more than women in cities who used osteopathy or yoga.	X		X	
Alderman & Kiepfer, 2003 [86]	Structured interviews	Psychiatry patients	52	4	51.9 % used CM in preceding 6 months. High use of nutritional supplements (66.7 %), 18.5 % visited a chiropractor. Drivers for use CM surrounded its usefulness with conventional treatment, natural healing & believed in CM philosophy.		X	X	X
Basedow et al., 2014 [46]	Cross sectional survey	Osteoarthritis patients	435	7	Females were more likely to use CM & > 70 years with a school education. 69 % reported CM use for disease management. 67 % CM users stated CM to be safe & 33 % felt it was effective in pain management.	X	X		X
Braun & Cohen, 2011 [59]	Cross sectional survey	Cardiac patients	161	4	No significant difference in age, gender, income or education between CM users & non-CM users. 51 % reported CM use. 71 % used CM to improve health, 30 % disease management, 20 % disease prevention.	X			X
Broom et al., 2012a [68]	Longitudinal study	Middle aged women	9,820	6	33 % consulted with a chiropractor & 40 % massage therapist. 63 % used CM & conventional practitioners. 2 % consulted with a CM practitioner only.			X	
Broom et al., 2012b [77]	Longitudinal survey	Middle aged women	10,492	6	42.4 % of women consulted with a CM practitioner. Women with back pain were more likely to use conventional therapy & CM (44.2 %). Women who consulted with a CM practitioner had better health compared to non-CM users.			X	
Brownie, 2006 [71]	Cross sectional survey	Elderly individuals	1,263	8	CM supplement use for arthritis, osteoporosis, hypertension & cardiovascular disease management. Females were more likely to report supplement use.	X			X
Buchbinder et al., 2002 [72]	Cross sectional survey	Rheumatoid arthritis patients	101	6	CM users more likely female & > 60 years. 73.3 % used CM, with 31.7 % consulting with a CM practitioner. 25.7 % used CM & conventional therapy for disease management.	X			X
Canaway & Manderson, 2013 [28]	Mixed methods	Diabetic patients with cardiovascular disease	2,766	9	CM users more likely to be > 50 years. 54.5 % reported consulting a CM practitioner & 45.1 % used CM regularly. 42.7 % believed in CM, 39.4 % believed CM was safe, 31.3 % used CM to control their health & 27.8 % preferred CM to other therapies.	X	X	X	X
Chatfield et al., 2009 [80]	Cross sectional survey	Ankylosing spondylitis patients	75	8	94.7 % CM users more likely female & have university education. 36 % CM users visited a massage therapist (81.5 %), acupuncture (6.7 %), naturopath (6.7 %) & homeopath (5.3 %).	X		X	X
Correa-Velez et al., 2003 [65]	Interviews (design not-specified)	Oncology patients	111	7	32 % were CM users with 56 % male & 44 % female, both with higher income. Most consulted practitioners were: reiki practitioner (33 %), a naturopath (27 %), or an integrative practitioner (27 %). 42 % used CM while participating in the study & 64 % CM use over last year.	X		X	X
Correa-Velez et al., 2005 [66]	Interviews (semi-structured)	Oncology patients	39	5	82 % of participants were regular CM users. Naturopathy (26 %), massage therapy (21 %) & integrative doctors (15 %) were the most common services used. CM used to survive cancer (67 %) & reduce cancer symptoms (33 %).		X	X	X
D’Onise et al., 2013 [31]	Cross sectional survey	General population	1,146	8	CM users were more likely to have a Bachelor degree, high gross household income, & full time employment. 32 % used CM products, 27 % used CM services. Individuals with chronic disease used CM products more than CM services 32.5 % vs 26.3 %. Services used were chiropractor (24.2 %), alternative therapy (5.4 %), & massage therapy (0.3 %).	X		X	X
Day, 2002 [82]	Cross sectional survey	Paediatric patients	92	4	No difference in age for CM users to non-CM users. 35.9 % used CM & 98.6 % were prepared to use CM.	X	X	X	
Day et al., 2004. [39]	Cross sectional survey	Children with Inflammatory bowel disease	46	4	Mean age of CM users was 11 years with 72 % being CM users. CM drivers related to dissatisfaction with standard care & advice from others. Homeopathy, chiropractic & massage consultations were used by <4 participants.	X	X	X	X
De Visser et al., 2000 [56]	Cross sectional survey	HIV/AIDS patients	894	5	56 % used CM. 45 % use both CM & conventional therapy. Women were more likely to only use CM. No other gender differences in CM use. Majority of CM users used nutritional & herbal supplements & massage therapy.	X	X	X	X
Dunning, 2003 [38]	Focus groups	Diabetic patients & practitioners	10	4	80 % were CM users. CM used for non-diabetic reasons. All participants used CM & conventional care for diabetes. Naturopathy & massage services were more likely used.			X	X
Edwards et al., 2014 [35]	Cross sectional survey	Oncology patients	639	5	Females had higher CM use (88.6 %). 82.9 % used CM during their cancer treatment with 56.3 % using manual therapies. CM users reported CM improved quality of life (42.6 %), supported health (33.6 %), managed cancer symptoms (26.2 %) & believe CM gave them hope.	X	X	X	X
Feldman & Laura, 2004 [44]	Cross sectional survey	University students	518	3	81.1 % used CM in the past 2 years. 82.5 % CM users female. Common treatments were relaxation techniques (41.7 %), massage therapy 38.2 %, herbal medicine (37.3 %), & art therapy (32.2 %). Drivers for CM were better results (34.5 %), lifestyle factors (33.1 %) & felt CM had fewer side effects (32.1 %).	X	X	X	
Field et al., 2008 [57]	Cross sectional survey	Women with high breast cancer risk	892	7	55 % reported CM use. 13.7 % used acupuncture, 28.2 % massage therapy, 12.3 % naturopathy & 7 % osteopathy. CM use was noted more in tertiary education & >50 year old individuals who resided in a major city.	X		X	
Fong & Fong, 2002 [43]	Cross sectional survey	Paediatric inpatients	120	3	33 % used CM. Massage therapies used by 17 %, 46 % naturopath, 29 % chiropractor & 10 % herbalist.		X	X	
Forster et al., 2006 [70]	Cross sectional survey	Pregnant women	588	8	36 % used herbal medicine during pregnancy. No identification of CM services used.	X			
Frawley et al., 2013 [29]	Longitudinal survey	Pregnant women	1,835	9	CM users were more likely to have a university degree, full time employment & higher income compared to non-CM users. 48.1% of women consulted with CM practitioners & 52 % used a CM product during pregnancy. Massage therapy was the most used 34.1 %, followed by chiropractic 16.3 %, acupuncture 0.6 %, naturopathy 7.2 %, osteopathy 6.1 % & doula services 1.4 %.	X		X	X
George et al., 2004 [60]	Cross sectional survey	Chronic obstructive pulmonary disease patients	173	4	41 % were CM users, mean age of 70 years. 55 % of CM users were male. CM used to promote health, reduce side effects & reduce disease progression.	X	X		X
Gollschewski et al., 2004 [87]	Cross sectional survey	Menopausal women	886	8	82.5 % CM users. CM users were middle aged (<55 years) & married. 66.8 % of women used nutritional supplements for menopausal management.	X			
Heath et al., 2012 [81]	Cross sectional survey	Palliative care in children with cancer	96	5	No significant difference in CM usage in terms of age, family income or education. 30 % used CM at end of life stage. 44 % reported using more than 1 CM therapy.	X			X
Hunter et al., 2014 [37]	Cross sectional survey	Radiotherapy patients	152	6	45.4 % CM users. Higher CM use in females & Caucasians. Young individuals more likely to use CM. CM users more likely to have secondary education & lower income. 2.9 % used acupuncture, 17.39 % chiropractor, 26.09 % massage therapy, 2.9 % osteopathy, 5.8 % naturopathy, 2.9 % Chinese Medicine & 1.45 % homeopathy. CM use was more likely in individuals diagnosed with breast, rectum, kidney, endometrium & skin cancers.	X		X	X
Klafke et al., 2012 [36]	Cross sectional survey	Male cancer patients	403	7	No difference in sociodemographic factors between CM users & non-CM users. 61.5 % used CM while undergoing cancer treatment.	X			X
Kremser et al., 2008 [53]	Cross sectional survey	Breast cancer patients	367	5	87.5 % used CM with 65.7 % CM users resided in NSW. CM use related to improving physical health (86.3 %), improving emotional health (86.3 %), supporting immune system (68.8 %), reducing side effects (49.2 %) and reducing the return of breast cancer (39.9 %). 41.4 % used massage therapy, 13.7 % acupuncture and 4.4 % naturopathy.	X		X	X
Leong et al., 2009 [84]	Cross sectional survey	Multiple sclerosis (MS) patients	428	5	66.3 % female & 60.3 % male participants used CM. Higher use in rural areas (70.4 %). 72.1 % used CM & conventional therapy for disease management.	X			X
Lim et al., 2005 [42]	Cross sectional survey	Children	503	6	51 % of children reported CM use with no difference in gender. Most common CM practitioners included 7 % chiropractic, 7 % aromatherapy, 5 % naturopathy, 5 % dietary & 5 % massage.	X		X	
MacLennan et al., 2006 [34]	Longitudinal study	General population	3,015	7	CM users were more likely 35–44 years. 29.3 % of women used CM services compared to males (23.6 %). 52.2 % used CM over the last year. Common practices included chiropractic 16.7 % & naturopathy 5.7 %. CM consultation higher in rural areas (29.4 %).	X		X	
Magin et al., 2006 [63]	Interviews (semi-structured)	Individuals with skin complaints	26	4	Most CM users were female. CM users felt CM was more efficacious than conventional medicine. Consultations were commonly with naturopaths & herbalists.	X	X	X	
Mak & Faux, t2010 [52]	Cross sectional survey	Osteoporotic patients	202	6	CM users were more likely female & 67 years old with post-secondary education. 51.5 % used CM for disease management. 19 % consulted with an acupuncturist, 12 % chiropractor/osteopathy, 6 % naturopathy & 2 % massage therapy. Drivers for CM used were holistic (53 %), reducing pain (29 %) & control over health (8.1 %).	X	X	X	X
Markovic et al., 2006 [51]	Cross sectional survey	Women with gynaecological cancers	53	4	17 % of women used CM. Most CM users were low income. Acupuncture was the only service used.	X	X	X	X
Murthy et al., 2014a [74]	Longitudinal study	Older aged women	1,310	9	Women in rural areas were more likely to use massage therapist. 76.4 % had a CM consultation with 41.4 % consulting with a massage therapist, 37.3 % chiropractor, 13.3 % acupuncture & 8.8 % osteopathy.	X		X	
Murthy et al., 2014b [95]	Longitudinal study	Older aged women	1,310	9	Sociodemographics were not associated with CM use. 75.2 % used self-prescribed CM products. Women were more likely to use CM treatments & a conventional practitioner.	X		X	
O’Callaghan & Jordan, 2003 [96]	Cross sectional survey	University students	171	4	CM used more likely female (77 %) & mean age of 29 years. 36.3 % CM users. 72 % consulted with a naturopath, 33 % aromatherapy & 31 % acupuncture.	X	X	X	
Patching van der Sluijs et al., 2007 [50]	Cross sectional survey	Menopausal women	1,296	5	53.8 % used CM services or products. 20.3 % consulted with a CM practitioner (7.2 % naturopath & 4.8 % acupuncture, were the most common).	X		X	
Rayner et al., 2009 [54]	Focus groups	Fertility clinic patients & practitioners	15	3	CM used for infertility due to a negative experience from assisted reproductive technologies or participants having a positive experience with CM.		X		X
Sarris et al., 2010 [76]	Cross sectional survey	Middle & older aged women	511	5	48 % consulted with a CM practitioner. Higher use of CM in 40–64 year olds (56.2 %). Older women consulted with a massage therapist or naturopath. Women who consulted with a practitioner had more health conditions.	X		X	
Shenfield et al., 2002 [58]	Structured interviews	Asthmatic children	174	2	51.7 % used CM in past year. 62.1 % currently use CM. 71.2 % used CM for preventative reasons & 17.5 % to improve asthma symptoms. 32 % visited a homeopath & 32 % a naturopath.		X	X	X
Shorofi & Arbon, 2010 [45]	Cross sectional survey	Hospitalised patients	353	5	90.4 % used CM, with women more likely to use CM. Services used were massage therapy (45 %), chiropractic (39.7 %), herbal medicine (38.2 %), & acupuncture (19.8 %). Rural CM users were more likely to use manual therapies compared to urban users who used biologically based therapies.	X	X	X	
Sibbritt et al., 2006 [79]	Longitudinal study	Middle aged women	11,143	9	16 % CM users consulted with a chiropractor &/or osteopathy were mid-aged. CM users were more likely rural residents & with school education only. Users were more likely to use CM with conventional medicine.	X		X	
Sibbritt et al., 2013 [62]	Longitudinal survey	Middle aged women	10,287	8	8.6 % of women used Chinese medicine. Users were more likely to have school education, born in Australia & live in rural or remote areas. Users were also more likely to frequently visit a doctor & Chinese medicine practitioner. Users also used other CM professionals including massage therapy (54 %), naturopathy (50 %), chiropractor (19 %), osteopathy (8 %) & acupuncture (47 %).	X		X	
Sinha & Efron, 2005 [40]	Cross sectional survey	Children with attention deficit hyperactivity disorder	75	4	67.6 % used CM for Attention deficit hyperactivity disorder. 58 % found CM helpful. CM use was associated with reducing side effects (67.4 %), hoping for a cure (66.7 %), reducing symptoms (88.9 %) & additional treatment to conventional therapy (69.7 %). 20 % visited a chiropractor.	X	X	X	
Skouteris et al., 2008 [97]	Cross sectional survey	Pregnant women	321	5	Sociodemographics were not different between CM users & non-CM users although CM users reported poorer health. 73.2 % reported CM use of which 29 % used CM for pregnancy related symptoms. 49.5 % consulted with a massage therapist & 5.9 % a naturopath	X		X	X
Smith & Eckert, 2006 [67]	Cross sectional survey	General population	2,985	7	18.4 % of children used CM. Most common consultations were chiropractic 34 %, massage therapy 21 %, & homeopathy 10.7 %. CM was used for preventing illness (39 %).	X		X	
Smith et al., 2013 [85]	Cross sectional survey	Female family planning patients	221	6	Younger women had less CM use compared to older women. 83 % of women report CM use, 33 % had consultations with a CM practitioner including chiropractic 12.4 %, acupuncture 11 % & 9.5 % naturopathy. CM users (49 %) viewed CM as having more natural benefit, (44 %) better alternative to conventional treatment, (38 %) as effective treatment & (36 %) gives individual control.	X	X	X	X
Spinks et al., 2014 [83]	Cross sectional survey	Diabetic patients with cardiovascular disease	2,915	8	Females were more likely to use CM & have a higher education & higher income. Chronic disease was associated with increased CM use. Women consulted with acupuncturists, naturopaths, nutritionists, chiropractors, & massage therapists.	X		X	X
Stankiewicz et al., 2007 [48]	Cross sectional survey	Infertility clinic patients	97	4	66 % CM users, 26 % used CM with conventional medicine. 48 % used CM services, most commonly acupuncture (9 %), naturopathy (17 %) & chiropractic (14 %).	X		X	X
Steel et al., 2012 [64]	Longitudinal study	Pregnant women	1,835	8	49.4 % consulted with a CM practitioner (massage therapy 34.1 %, 16.3 % chiropractor were more common). 22.2 % consulted with both a CM & conventional practitioners.			X	X
Steel et al., 2014a [98]	Longitudinal survey	Pregnant women	1,835	9	Women in non-urbans areas were more likely to consult a chiropractor. Women felt CM promoted holistic health & reduced conventional side effects. 53 % of women who used non-pharmacological pain management used a CM practitioner or products (49 %).	X	X	X	X
Steel et al., 2014b [99]	Longitudinal survey	Pregnant women	2,445	9	Chiropractor users were more likely located in non-urban areas & have permanent employment. 49.4 % consulted with a CM practitioner. 74.4 % used non-pharmacological pain management. 60.7 % used CM products or services. 80.7 % consulted with a practitioner. CM users believed CM had fewer side effects & was more natural & offered more control compared to conventional treatment.	X	X	X	X
Trutnovsky et al., 2001 [78]	Cross sectional survey	Sexual health clinic patients	63	4	59 % – 96 % CM use, depending on condition. CM users more likely to be female.	X		X	
Wadhera et al., 2011 [41]	Cross sectional survey	Children	98	6	No difference between CM users & non-CM users regarding age, gender & illness. 67 % used CM previously or currently. 70 % used CM for disease management. Drivers for use surrounded dissatisfaction with conventional treatment, belief in CM, reduce side effects & lack of suitable conventional treatment.	X	X	X	
Wilkinson & Simpson, 2001 [32]	Cross sectional survey	Rural residents	300	7	Females were more likely to consult & use CM products. 62.7 % consulted with a CM practitioner. 70.3 % use some form of CM. 68.7 % used CM products. Chiropractors consulted 55.3 %. 56.2 % felt CM improved quality of life.	X	X	X	
Wilkinson & Jelinek, 2009 [33]	Cross sectional survey	Rural residents	102	5	There was no difference in gender & CM services used. 78 % used CM therapies, 66 % consulted with a CM practitioner (15 % naturopathy, 17 % massage, 17 % chiropractic). Drivers with CM use were positive attitudes towards CM, holism, anti-science, individual responsibility & rejection to authority.	X	X	X	
Xue et al., 2007 [30]	Cross sectional survey	General population	1,067	8	71.2 % were CM users & identified as females, higher income earners & having a higher education. 16.4 % visited a clinical nutritionist, 73.7 % massage therapy, 29.1 % Western herbal medicine & 90.6 % chiropractor.	X		X	

A number of significant gaps were identified from the risk of bias assessment. A large number of gaps were noted in the external validity criteria, particularly in relation to representativeness of the study to the national population, a true representation of the target population and the likelihood that nonresponse bias was minimal. Internal validity assessment highlighted gaps relating to inclusion of an appropriate prevalence period, appropriate parameters of the numerator and dominator of interest and inclusion of an overall risk of bias summary on the study. A majority of these gaps were identified from poor or inadequate reporting in the selected articles. Results from the risk of bias assessment is displayed in Table 3.

**Table 3:** Risk of bias assessment of the selected articles

Risk of bias assessment	External validity				Internal validity							Score
	Representativeness to national population	True representation of the target population	Random sampling methods	Likelihood of nonresponse bias minimal	Data directly collected from participants	Acceptable case definition	Validated study tool used	Consistent data collections methods	Appropriate prevalence period	Appropriate parameters of numerator & denominator	Summary of overall risk of study bias	
Adams et al., 2003 [27]	X	X	X	X	X	X	X	X	X	X		10
Adams et al., 2005 [75]	X	X	X	X	X	X		X	X	X		9
Adams et al., 2007 [47]	X	X	X	X	X		X	X	X	X		9
Adams et al., 2011 [49]	X	X	X	X	X			X	X	X		8
Adams et al., 2012 [55]	X	X	X	X	X		X	X	X	X	X	10
Adams et al., 2013 [73]	X	X	X	X	X			X	X	X	X	9
Alderman & Kiepfer, 2003 [86]					X			X	X			3
Basedow et al., 2014 [46]			X		X		X	X		X		5
Braun & Cohen, 2011 [59]					X	X		X		X	X	5
Broom et al., 2012a [68]	X	X	X	X	X		X	X	X	X	X	10
Broom et al., 2012b [77]	X	X	X	X	X		X	X	X	X	X	10
Brownie, 2006 [71]		X	X		X			X		X		5
Buchbinder et al., 2002 [72]			X	X	X	X		X	X		X	7
Canaway & Manderson, 2013 [28]			X		X	X	X	X	X	X		7
Chatfield et al., 2009 [80]					X	X		X	X	X		5
Correa-Velez et al., 2003 [65]		X		X	X	X	X	X		X		7
Correa-Velez et al., 2005 [66]					X			X				2
D’Onise et al., 2013 [31]	X	X	X	X	X	X	X	X	X	X	X	11
Day, 2002 [82]						X		X		X		3
Day et al., 2004 [39]					X							1
De Visser et al., 2000 [56]					X		X	X				3
Dunning, 2003 [38]					X			X				2
Edwards et al., 2014 [35]		X		X	X		X	X		X		6
Feldman & Laura, 2004 [44]					X			X				2
Field et al., 2008 [57]		X			X			X			X	4
Fong & Fong, 2002 [43]								X		X		2
Forster et al., 2006 [70]				X	X		X	X		X		5
Frawley et al., 2013 [29]	X	X	X	X	X	X		X	X	X	X	10
George et al., 2004 [60]					X	X		X				3
Gollschewski et al., 2004 [87]		X	X		X			X		X		5
Heath et al., 2012 [69]								X		X	X	3
Hunter et al., 2014 [37]		X			X			X		X	X	5
Klafke et al., 2012 [36]		X			X	X		X		X		5
Kremser et al., 2008 [53]					X			X			X	3
Leong et al., 2009 [84]		X			X			X			X	4
Lim et al., 2005 [42]						X		X	X	X		4
MacLennan et al., 2006 [34]	X	X	X	X	X			X	X	X	X	9
Magin et al., 2006 [63]					X			X				2
Mak & Faux, 2010 [52]		X	X		X	X		X				5
Markovic et al., 2006 [51]					X	X		X				3
Murthy et al.,2014a [74]	X	X	X	X	X			X	X	X	X	9
Murthy et al., 2014b [95]	X	X	X	X	X			X	X	X	X	9
O’Callaghan & Jordan, 2003 [96]					X			X				2
Patching van der Sluijs, et al., 2007 [50]					X			X	X	X	X	5
Rayner et al., 2009 [54]					X			X				2
Sarris et al., 2010 [76]		X			X			X	X			4
Shenfield et al., 2002 [58]		X						X				2
Shorofi & Arbon, 2010 [45]		X			X			X		X		4
Sibbritt et al., 2006 [79]	X	X	X	X	X		X	X	X	X		9
Sibbritt et al., 2013 [62]	X	X	X	X	X		X	X	X	X		9
Sinha & Efron, 2005 [40]		X						X		X		3
Skouteris et al., 2008 [97]		X			X			X	X		X	5
Smith & Eckert, 2006 [67]		X		X	X			X	X	X		6
Smith et al., 2013 [85]		X			X			X	X	X	X	6
Spinks et al., 2014 [83]	X	X	X		X		X	X	X	X	X	9
Stankiewicz et al., 2007 [48]		X			X			X	X		X	5
Steel et al., 2012 [64]	X	X	X	X	X			X	X	X	X	9
Steel et al., 2014a [98]	X	X	X	X	X			X	X	X	X	9
Steel et al., 2014b [99]	X	X	X	X	X			X	X	X	X	9
Trutnovsky et al., 2001 [78]					X			X				2
Wadhera et al., 2011 [41]		X		X		X		X		X		5
Wilkinson & Simpson, 2001 [32]			X		X			X	X			4
Wilkinson & Jelinek, 2009 [33]					X		X	X	X			4
Xue et al., 2007 [30]	X	X	X		X			X	X		X	7

### Sociodemographic characteristics of CM users

Correlation between adult CM use and gender was identified in a number of articles, reporting a higher rate of CM consumption amongst female CM users compared to male CM users in general population based studies [30–34]. In these studies, female CM users were more likely to be middle-aged, have a higher education level and a higher annual income, compared to female non-CM users. Ethnicity was also a key characteristic of CM use and was found to be higher in Caucasian populations [30, 33, 35–38]. In comparison there was no difference in age, gender or disease status of children who used CM products or CM services [39–42] however one study reported higher CM use by children whose parents had a higher education or a managerial occupation and used CM themselves [43].

Individuals residing in rural areas were more likely to utilise CM in general [37, 44] and in particular manual therapies [34, 45] when compared to individuals in urban localities. Individuals in remote, outer regional and inner regional areas are more likely to consult with chiropractors compared to individuals in major cities [34] with 55.3 % of the population in rural NSW reporting use of chiropractic services [46]. The impact of locality on naturopathic consultations is not as clear with some studies reporting increased consultation rates in non-urban areas (63 %) [47] and others identifying lower consultation rates (15 %–31.4 %) in rural areas [46, 48].

### Drivers of CM use

Over half of the selected articles identified various ‘push’ and ‘pull’ factors as drivers behind CM use. Patient interactions and experiences with the conventional health system appear influential with unsatisfactory results from conventional therapy [38, 47, 49–52], and the desire to further reduce symptoms or side effects from conventional therapy [27, 38, 41, 50, 51, 53, 54] both being reported as popular drivers of CM use. Patients were also drawn positively to CM for a number of other reasons including: attraction to the perceived notion of CM as a holistic method of health care [55, 56]; the ability to use CM as a preventive therapy [27, 50, 53, 57, 58]; and the therapeutic value of CM as an adjunctive therapy to conventional medicine [50, 53]. Other drivers amongst patients using CM centred on the perceived alignment of CM with the individual’s personal belief system [59], the perception of CM as safe [60], the ability for CM to provide hope [61] or a sense of patient control over their treatment [50, 62, 63], and a perception that CM practitioners are more supportive towards their health compared to other health professionals [27, 64]. Within subpopulations with chronic health conditions, CM use was linked to reducing side effects from conventional medicine, dissatisfaction with standard care and to assist in disease management [28, 39, 41, 46, 53, 56, 61, 65, 66].

### Use of health services by CM users

CM users were identified as accessing multiple health services from a wide variety of conventional and CM disciplines. CM users appear to be higher users of conventional medical care, with several articles reporting CM users as visiting general practitioners more frequently than non-CM users [32, 44, 47, 49, 67]. The majority of CM services used for a diversity of conditions were chiropractic, massage therapy, naturopathy and acupuncture [29, 30, 33, 34, 42, 44, 45, 48–50, 53, 55, 57, 63–66, 68–78]. Results indicate that chiropractors and massage therapists are the most commonly consulted CM disciplines, with chiropractor consultations reported up to 55.3 % [32], with a higher use by rural residents [33, 79] and male CM users [32]. Massage therapy consultations were reported by up to 81.5 % [80] amongst those with musculoskeletal complaints. Rates of acupuncture consultations were reported to range from 6.7 % and 32.2 %, with a higher rate of use amongst pregnant women [64] and increased frequency of use in fertility clinic patients [48]. The least commonly consulted CM practitioner across all populations were homeopaths [46, 68].

### CM use amongst health subpopulations

CM use was reported in a number of chronic diseases including those identified as Australian NHPA. Patients accessed CM to assist in the management of a number of chronic diseases including: cancer [53, 65, 75, 81]; musculoskeletal diseases (rheumatoid arthritis, osteoporosis, osteoarthritis and ankylosing spondylitis) [44, 46, 49, 52, 72, 80]; digestive disease (inflammatory bowel disease) [39, 82]; asthma [58, 61]; cardiovascular diseases [83]; multiple sclerosis [84]; diabetes mellitus [28, 38, 83]; mental health [32, 44, 47, 49, 55, 85, 86]; and HIV [56]. CM use for chronic disease management was associated with users reporting poorer health compared to non-CM users [27] and evidence of utilising both CM and conventional professionals [75]. Alongside individuals with chronic health conditions, pregnant women also used CM concurrent to conventional maternity care with almost half of pregnant women consulting specifically with a CM practitioner for pregnancy-related complaints [64]. In addition, two studies reported high CM use (53.8 %–82.5 %) by women experiencing menopause-associated symptoms [50, 87].

## Discussion

This is the first critical review of a large body of research which has explored the contemporary nature of CM use in Australia. This review identifies high use of CM in line with international sociodemographic trends including the predominance of females [12] and those with tertiary education qualifications [30, 37]. A number of reasons to explain the association between education level and CM use have been suggested to include: higher levels of health literacy and access to resources [32, 88]; potential for self-determination [16]; and greater disposable income to spend on healthcare [89]. If these reasons apply to Australian CM users, it may suggest such users are potentially conducting their own research to inform self-determined health choices. The relationship between CM use and level of education is of particular interest in Australia given the past and current concerns about CM use by the peak scientific body [5] which appear to go unheeded by members of the population with higher education. Exceptions to the identified relationship between higher education level and CM use is apparent amongst older adults where post-secondary education is less prominent [90]. This difference may be explained by the higher rates of chronic disease in this population [91], however, less is known about the factors which influence this group [92]. As such, more research is needed to better understand health decision-making amongst older adults with chronic disease.

Our review identifies individuals with chronic diseases or co-morbidities, and a lowered quality of life, have a higher reported CM health service utilisation when compared with non-CM users. Many of the mentioned chronic diseases, such as cancer, musculoskeletal diseases, mental health and diabetes, are identified as NHPA by the Australian Government due to their high mortality and morbidity rates in the population [8]. Given the priority focus on these diseases, the higher rates of CM use by individuals with these conditions requires further research and policy attention. Despite the trends identified in this review, very little is known about CM use and users within chronic disease subpopulations. In particular a more detailed description of the specific CM used by individuals with chronic disease including the reasons for use, their concurrent use of conventional treatments, and the effectiveness and safety of CM as part of their overall health care is urgently needed. Alongside this, the interprofessional dynamics between CM and conventional health professionals providing care to the same individual is an important area requiring further research focus, given that amongst chronic disease subpopulations CM users are also more likely than non-CM users to have an increased frequency of consultations with their general practitioner and/or allied health professional [27, 47, 49, 55, 68].

This review identified key differences in CM services in rural and non-urban areas, compared to urban populations. In particular, the use of manual therapies such as consultations with a massage therapist or chiropractor were more common amongst rural populations. These geographical insights suggests there may be other more specific drivers or influences of CM use in rural areas [49]. It has been proposed that access to both CM and conventional health services and overall CM workforce distribution may be influential in the differences in CM use in rural areas when compared with urban populations [15]. In addition, rural CM users are more likely to have a lower household income compared with their urban counterparts [33]. The reasons underpinning this economic characteristic require further clarification. Overall, the higher rate of CM use and contrasting profile of users of CM in rural areas deserves close research and policy attention given the important challenges facing rural health care at a federal and state government level [93, 94] in Australia.

### Review limitations and future areas of research

A number of gaps in the reviewed literature were identified in relation to study quality and findings. Study quality could be improved by using a representative sampling method of the general population in order to gain a greater view of CM use in the general population. As a number of studies were conducted in individual states, additional data from other states is needed to provide a more complete picture of CM use in Australia. In addition the gaps identified in the risk of bias assessment may pose as an issue for the strength of the results descripted in this review.

As mentioned, CM users are more likely to be female and in line with this more studies have been conducted to explore female CM users and use in Australia. Studies targeting male-specific populations and CM use are recommended for future studies. An additional priority area is a thorough examination of effectiveness, economics, and safety of the CM being used to manage chronic disease. To adequately address all of these identified areas comprehensive research designs which examine CM within the context of chronic disease is paramount.

Heterogeneity in research design and methodology may limit the ability to draw broader conclusions about CM use from this review. Of significance is the absence of definition for CM across the contemporary literature. This is an important issue due to widely recognised need for a uniform definition of CM and a lack of clarity regarding which professions and practices fit under the umbrella term. Nevertheless, this review does provide the first summative critical review of research examining the nature of CM use in Australia providing important insights for both health services research as well as practice and policy development around CM use in Australia.

## Conclusion

CM use is substantial across contemporary Australia and all involved in managing, organising, providing and using health care services in Australia need to be cognisant of CM use, especially as concurrent to conventional medicine use and consultation with conventional health care providers. Further research examining a range of identified areas around CM use in Australia will help contribute to wider practice and policy development and attempts to provide effective, safe and coordinated health care for all Australians.

### Abbreviations

CM, Complementary medicine; DOHA, Department of Health and Aging; HIV, Human Immunodeficiency Virus; NHMRC, National Health and Medical Research Council; NHPA, National Health Priority Areas; NSW, New South Wales; UK, United Kingdom; USA, United States of America
